# Supplementary material for: Inhibition of lung tumorigenesis by a small molecule CA170 targeting the immune checkpoint protein VISTA
Source: Commun Biol. 2021 Jul 23;4:906. doi: 10.1038/s42003-021-02381-x (PMC8302676; doi:10.1038/s42003-021-02381-x)
Supplement: Supplementary file 2 — Supplementary Information [file 42003_2021_2381_MOESM2_ESM.pdf]

Supplementary information

Title:

Inhibition of lung tumorigenesis by a novel small molecule CA170 targeting the immune checkpoint protein VISTA

Authors:

Jing Pan[1,2,3*], Yao Chen[4,5*], Qi Zhang[1,2,3*], Achia Khatun[4,5], Katie Palen[6], Gang Xin[4,5], Li Wang[7], Chuanjia Yang[1,2], Bryon D. Johnson[6], Charles R. Myers[1,2], Shizuko Sei[8], Robert H. Shoemaker[8], Ronald A. Lubet[8], Yian Wang[1,2,3], Weiguo Cui[4,5#], Ming You[1,2,3#]

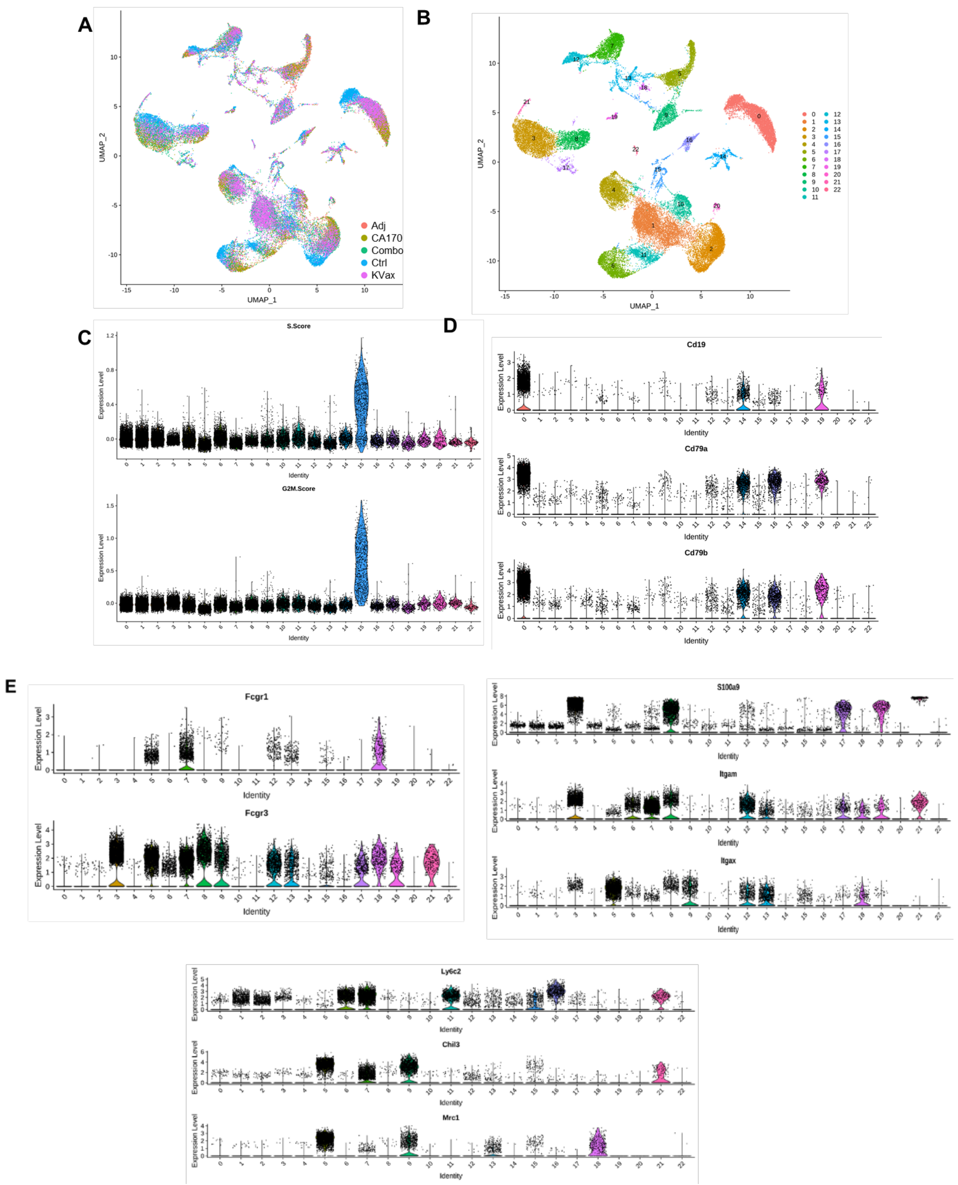

Supplementary Figure 1: Cellular heterogeneity of total immune cells for the five different lung cancer treatments. (A) The UMAP plot shows the overlap of total immune cells for the five lung cancer treatments. (B) Division of total immune cells into 22 different clusters based on 22 different principle components (PCs) for the five different treatment conditions. (C) Violin plot showing scoring of cell cycle genes across 22 different clusters for all immune cells for the five treatment conditions. (D and E) Violin plots showing B cell and myeloid cell specific markers across 22 different clusters in total immune cells for the five different lung cancer treatments.

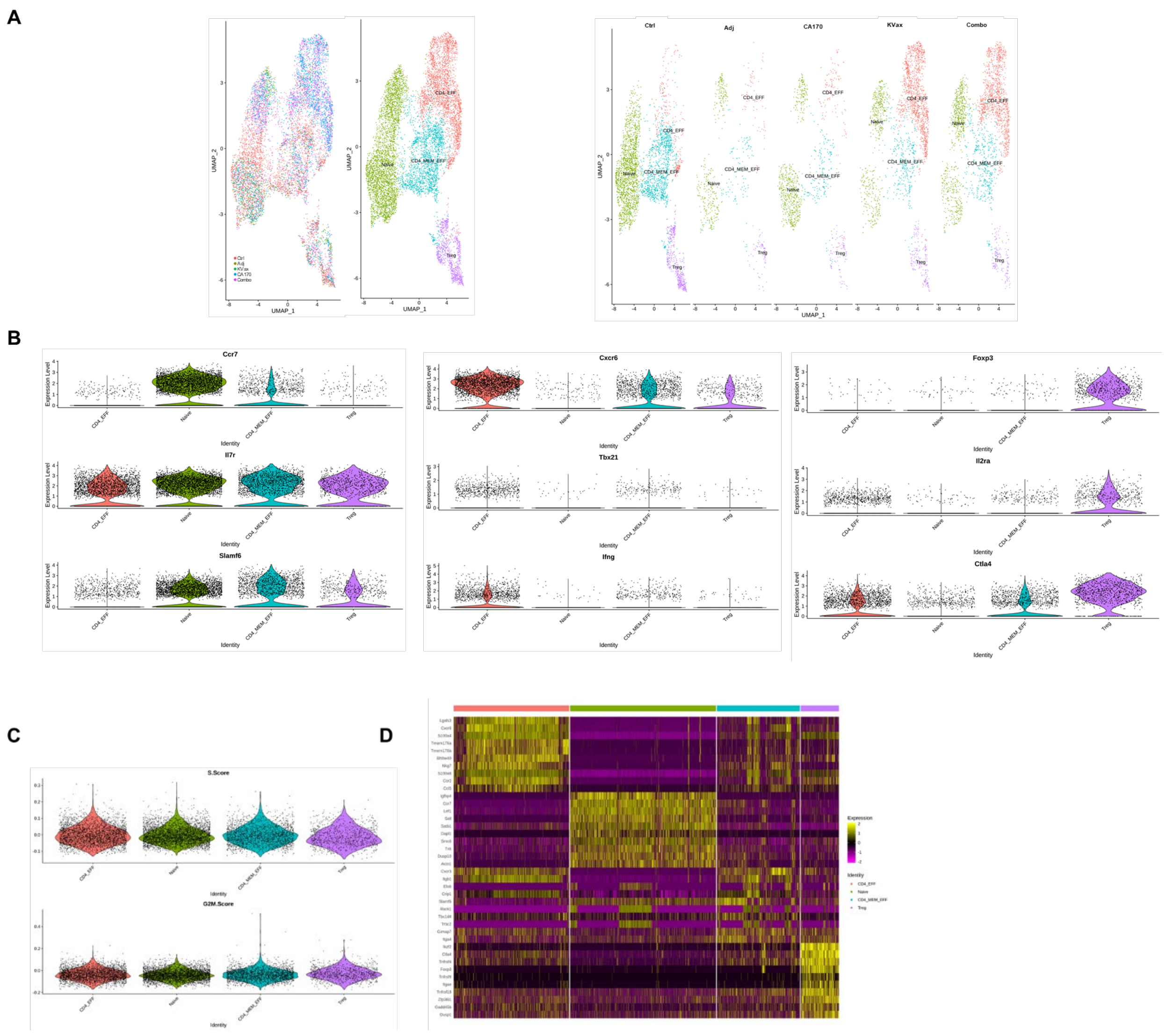

Supplementary Figure 2: Heterogenous CD4+ T cells for the five different lung cancer treatments. (A) UMAP plot shows overlap of all CD4+ T cells in four CD4+ T cell samples (Left), and the distribution of different CD4+ T cell subsets individually (Right) across different lung cancer treatments. (B) Violin plots show expression of naïve CD4 T cell specific genes (Left), CD4+ Th1 specific effector genes (Middle), Treg specific genes (Right) and cell cycle specific genes (C) across different CD4+ T cells clusters from the combined CD4+ T cell data under five treatments. (D) Heatmap showing expression of the top 10 genes for each of the clusters from the combined CD4+ T cell data for the five treatments.

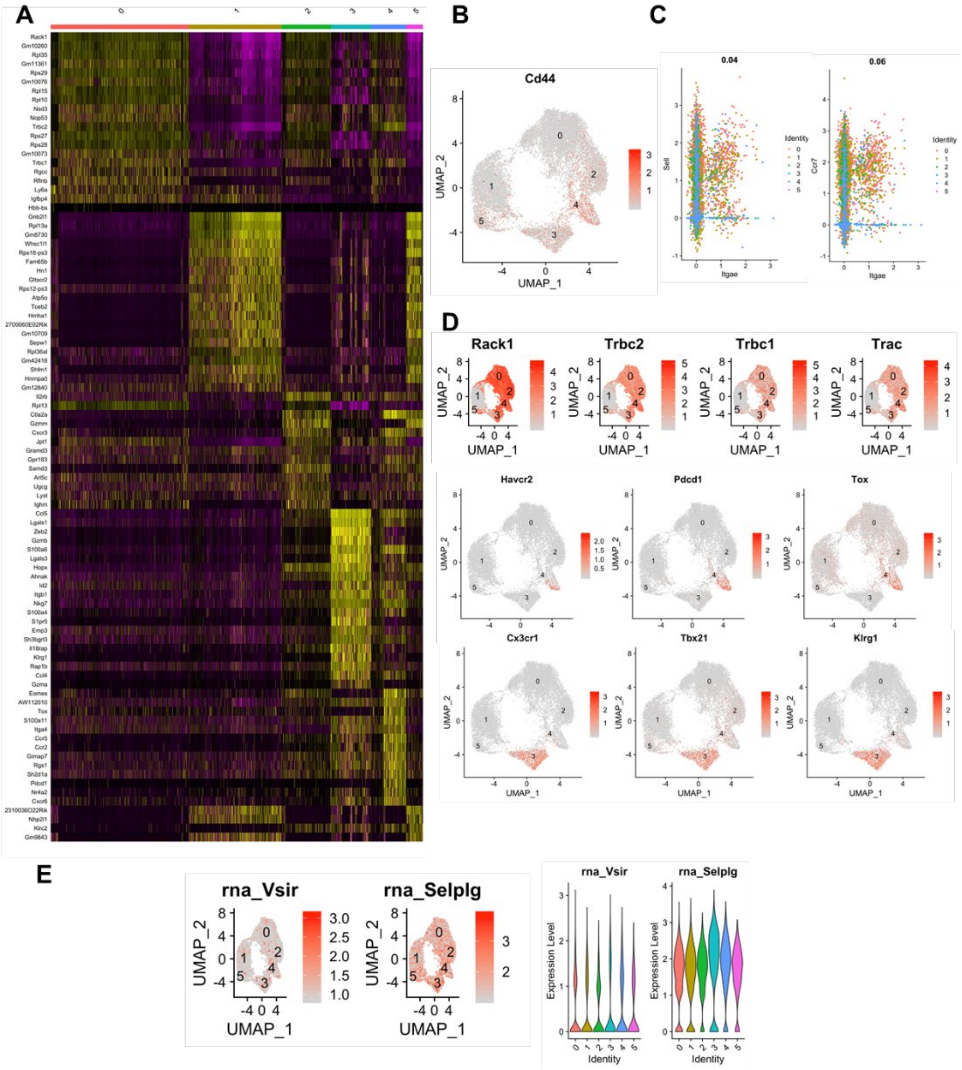

Supplementary Figure 3: Heterogenous CD8+ T cells for the five different lung cancer treatments. (A) Heatmap showing expression of the top 20 markers of the six CD8+ T cell clusters. (B) Feature plot shows expression of Cd44 in the 6 CD8+ T cell clusters. (C) Scatter plot shows the co-expression of Sell/Ccr7 with Itgae in each cell. Cells are colored by their cluster. Pearson correlation between the two features is displayed above the plot. (D) Feature plots show expression of signatures genes of the 6 CD8+ T cell clusters. (E) Feature plots and violin plots show expression of VISTA receptor and ligand for the 6 CD8+ T cell clusters.

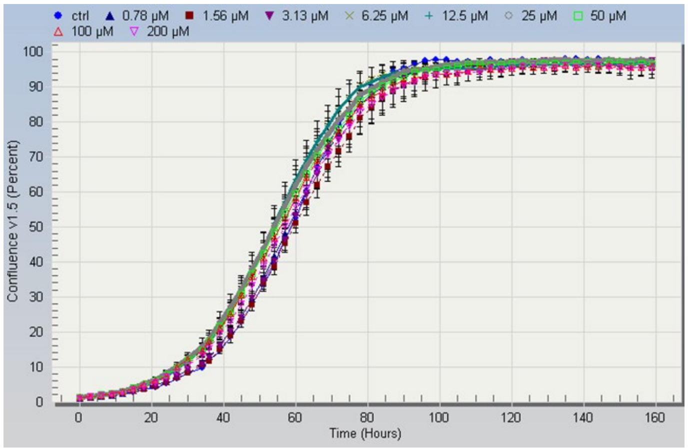

Supplementary Figure 4: CA170 does not direct kill mouse lung cancer cells when treated with up to 200 μM for 6 days. Dose-response effects of CA170 on LKR13 cell proliferation by real time monitoring cell confluences using Incucyte. Data are shown as the mean ± SE, n=3.
