# Supplementary material for: Inhibition of lung tumorigenesis by a small molecule CA170 targeting the immune checkpoint protein VISTA
Source: Commun Biol. 2021 Jul 23;4:906. doi: 10.1038/s42003-021-02381-x (PMC8302676; doi:10.1038/s42003-021-02381-x)
Supplement: Supplementary file 3 — Description of Supplementary Files [file 42003_2021_2381_MOESM3_ESM.pdf]

Description of Additional Supplementary Files

File name: Supplementary Data 1

Description: The source data underlying all Figures.
