# Supplementary material for: Inhibition of lung tumorigenesis by a small molecule CA170 targeting the immune checkpoint protein VISTA
Source: Commun Biol. 2021 Jul 23;4:906. doi: 10.1038/s42003-021-02381-x (PMC8302676; doi:10.1038/s42003-021-02381-x)
Supplement: Supplementary file 5 — Reporting Summary [file 42003_2021_2381_MOESM5_ESM.pdf]

nature research

Corresponding author(s): DBPR: COMMSBIO-20-1459-T

Last updated by author(s): Jun 4, 2020

Reporting Summary

Nature Research wishes to improve the reproducibility of the work that we publish. This form provides structure for consistency and transparency in reporting. For further information on Nature Research policies, see our Editorial Policies and the Editorial Policy Checklist.

Statistics

For all statistical analyses, confirm that the following items are present in the figure legend, table legend, main text, or Methods section.

n/a	Confirmed	
☐	☒	The exact sample size (*n*) for each experimental group/condition, given as a discrete number and unit of measurement
☐	☒	A statement on whether measurements were taken from distinct samples or whether the same sample was measured repeatedly
☐	☒	The statistical test(s) used AND whether they are one- or two-sided<br>*Only common tests should be described solely by name; describe more complex techniques in the Methods section.*
☐	☒	A description of all covariates tested
☐	☒	A description of any assumptions or corrections, such as tests of normality and adjustment for multiple comparisons
☐	☒	A full description of the statistical parameters including central tendency (e.g. means) or other basic estimates (e.g. regression coefficient) AND variation (e.g. standard deviation) or associated estimates of uncertainty (e.g. confidence intervals)
☒	☐	For null hypothesis testing, the test statistic (e.g. *F*, *t*, *r*) with confidence intervals, effect sizes, degrees of freedom and *P* value noted<br>*Give P values as exact values whenever suitable.*
☒	☐	For Bayesian analysis, information on the choice of priors and Markov chain Monte Carlo settings
☒	☐	For hierarchical and complex designs, identification of the appropriate level for tests and full reporting of outcomes
☒	☐	Estimates of effect sizes (e.g. Cohen's *d*, Pearson's *r*), indicating how they were calculated

Our web collection on statistics for biologists contains articles on many of the points above.

Software and code

Policy information about availability of computer code

Data collection	All flow cytometry data were acquired on an BD LSR-II (BD Biosciences, CA). The 10X Genomics Chromium Controller was used to generate a single cell RNA-seq library.
Data analysis	All flow cytometry data were analyzed using FlowJo (Treestar, OR). Statistical tests were performed using Graphpad Prism 8. Raw sequencing data were demultiplexed and converted to gene-barcode matrices using the Cell Ranger (version 2.2.0) mkfastq and count functions, respectively (10x Genomics). The mouse reference genome mm10 was used for alignment. Data were further analyzed in R (version 3.4.0) using Seurat (version 3).

For manuscripts utilizing custom algorithms or software that are central to the research but not yet described in published literature, software must be made available to editors and reviewers. We strongly encourage code deposition in a community repository (e.g. GitHub). See the Nature Research guidelines for submitting code & software for further information.

Data

Policy information about availability of data

All manuscripts must include a data availability statement. This statement should provide the following information, where applicable:

- Accession codes, unique identifiers, or web links for publicly available datasets
- A list of figures that have associated raw data
- A description of any restrictions on data availability

Tumor-infiltrating CD45+ cells rep_1, GEO Accession codes will be available soon
Tumor-infiltrating CD45+ cells rep_2, GEO Accession codes will be available soon
Tumor-infiltrating CD3+ cells rep_1, GEO Accession codes will be available soon
Tumor-infiltrating CD3+ cells rep_2, GEO Accession codes will be available soon

Figure 5, Tumor-infiltrating CD45+ cells rep_1 and Tumor-infiltrating CD45+ cells rep_2
Figure 6, Tumor-infiltrating CD45+ cells rep_1, Tumor-infiltrating CD45+ cells rep_2, Tumor-infiltrating CD3+ cells rep_1 and Tumor-infiltrating CD3+ cells rep_2
Figure 7, Tumor-infiltrating CD45+ cells rep_1, Tumor-infiltrating CD45+ cells rep_2, Tumor-infiltrating CD3+ cells rep_1 and Tumor-infiltrating CD3+ cells rep_2

Both raw data and processed data of single-cell RNA-seq data will be deposited in the GEO database with the accession code.

Field-specific reporting

Please select the one below that is the best fit for your research. If you are not sure, read the appropriate sections before making your selection.

☒ Life sciences ☐ Behavioural & social sciences ☐ Ecological, evolutionary & environmental sciences

For a reference copy of the document with all sections, see nature.com/documents/nr-reporting-summary-flat.pdf

Life sciences study design

All studies must disclose on these points even when the disclosure is negative.

Sample size	Sample size were calculated based on the detectable differences with at least 80% power, based on a two-sided t-test or chi-square test with a Bonferroni adjusted significance level of 0.05/3=0.017 to protect against multiple testing.
Data exclusions	No data was excluded from the analysis.
Replication	All flow cytometry experiments were replicated successfully for three time. While single cell RNA-seq was only conducted once.
Randomization	Animals were randomized to treatment groups based on mean body weight measurements, ensuring no statistical differences between animals at baseline.
Blinding	No blinding was used.

Reporting for specific materials, systems and methods

We require information from authors about some types of materials, experimental systems and methods used in many studies. Here, indicate whether each material, system or method listed is relevant to your study. If you are not sure if a list item applies to your research, read the appropriate section before selecting a response.

Materials & experimental systems

n/a	Involved in the study	
☐	☒	Antibodies
☐	☒	Eukaryotic cell lines
☒	☐	Palaeontology and archaeology
☐	☒	Animals and other organisms
☒	☐	Human research participants
☒	☐	Clinical data
☒	☐	Dual use research of concern

Methods

n/a	Involved in the study	
☒	☐	ChIP-seq
☐	☒	Flow cytometry
☒	☐	MRI-based neuroimaging

Antibodies

Antibodies used

ANTIBODY COMPANY CAT #
CD3 APC eFluor780 ThermoFisher 47-0032-82
Ly-6C PE BD Biosciences 560592
7AAD BD Biosciences 559925
CD45 BV786 BD Biosciences 564225
CD8 BUV396 BD Biosciences 563786
Ly-6G BUV396 BD Biosciences 563978
GRANZYME B AF647 Biolegend 515406
CD4 FITC ThermoFisher 11-0042-82
CD25 PE ThermoFisher 12-0251-82
FOXP3 eFluor450 ThermoFisher 48-5773-82
CD62L APC ThermoFisher 17-0621-82
CD44 PeCy7 ThermoFisher 25-0441-82
CD19 SB600 ThermoFisher 63-0193-82
CD11b eFluor450 ThermoFisher 48-0112-82
CD11c APC ThermoFisher 17-0114-82
IFN-g PE ThermoFisher 12-7311-82

	TNFa PeCy7 ThermoFisher 25-7321-82
Validation	All flow cytometry antibodies used in this study are commercially available, and they were used only on species for which they have been validated by the vendor.

Eukaryotic cell lines

Policy information about cell lines

Cell line source(s)	LKR13 cells originated from KrasLA1 mice, and express mutant KrasG12D on the sv129 background, were a generous gift from Dr. Jonathan M. Kurie (MD Anderson).
Authentication	The cell line was not authenticated.
Mycoplasma contamination	Cells were tested negative for mycoplasma contamination.
Commonly misidentified lines (See ICLAC register)	*Name any commonly misidentified cell lines used in the study and provide a rationale for their use.*

Animals and other organisms

Policy information about studies involving animals; ARRIVE guidelines recommended for reporting animal research

Laboratory animals	A/J mice from Jackson Laboratories are used in this study.
Wild animals	*Provide details on animals observed in or captured in the field; report species, sex and age where possible. Describe how animals were caught and transported and what happened to captive animals after the study (if killed, explain why and describe method; if released, say where and when) OR state that the study did not involve wild animals.*
Field-collected samples	*For laboratory work with field-collected samples, describe all relevant parameters such as housing, maintenance, temperature, photoperiod and end-of-experiment protocol OR state that the study did not involve samples collected from the field.*
Ethics oversight	All procedures were in accordance with the Medical College of Wisconsin Institutional Animal Care and Use Committee.

Note that full information on the approval of the study protocol must also be provided in the manuscript.

Flow Cytometry

Plots

Confirm that:

☒ The axis labels state the marker and fluorochrome used (e.g. CD4-FITC).

☒ The axis scales are clearly visible. Include numbers along axes only for bottom left plot of group (a 'group' is an analysis of identical markers).

☒ All plots are contour plots with outliers or pseudocolor plots.

☒ A numerical value for number of cells or percentage (with statistics) is provided.

Methodology

Sample preparation	For immune profiling, tumors were harvested and pooled from each mouse at the end of study, minced into 1-2 mm pieces and digested at 37oC for 20 min with mouse tumor dissociation buffer (MiltenyiBiotec, CA) to generate single cell suspensions per the manufacturer's instructions. Tumor-infiltrating leukocytes were directly stained for flow cytometry sorting or analysis.
Instrument	LSR Fortessa™ X-20 or LSR-II flow cytometer (Becton Dickinson)
Software	Flowjo version 10
Cell population abundance	Sorted Tils were reanalyzed by flow cytometry to ensure the purity of the cell population.
Gating strategy	Size>Live>CD45+>CD3+>CD4+ or CD8+ >CD44/CD62L;<br>Size>Live>CD45+>CD3+>CD4+>FoxP3/CD25;<br>Size>Live>CD45+>CD3->CD11c->CD11b+>Ly6G/Ly6c;<br>Size>Live>CD45+>CD3+>CD4+ or CD8+ >IFNg/TNFa/GranzymeB;

☐ Tick this box to confirm that a figure exemplifying the gating strategy is provided in the Supplementary Information.
